# Phenotypes of a *Pseudomonas aeruginosa* hypermutator lineage that emerged during prolonged mechanical ventilation in a patient without cystic fibrosis

**DOI:** 10.1128/msystems.00484-23

**Published:** 2023-12-22

**Authors:** Sophia H. Nozick, Egon A. Ozer, Rachel Medernach, Travis J. Kochan, Rebecca Kumar, Jori O. Mills, Richard G. Wunderlink, Chao Qi, Alan R. Hauser

**Affiliations:** 1 Department of Microbiology-Immunology, Northwestern University Feinberg School of Medicine, Chicago, Illinois, USA; 2 Department of Medicine, Division of Infectious Diseases, Northwestern University Feinberg School of Medicine, Chicago, Illinois, USA; 3 Center for Pathogen Genomics and Microbial Evolution, Robert J. Havey Institute for Global Health, Northwestern University Feinberg School of Medicine, Chicago, Illinois, USA; ^4^Department of Medicine, Division of Infectious Diseases, Georgetown University, Washington, DC, USA; 5 Department of Medicine, Division of Pulmonary and Critical Care, Northwestern University Feinberg School of Medicine, Chicago, Illinois, USA; 6 Department of Pathology, Northwestern University Feinberg School of Medicine, Chicago, Illinois, USA; North Carolina Agricultural and Technical State University, Greensboro, North Carolina, USA

**Keywords:** *Pseudomonas aeruginosa*, hypermutator, genomics, virulence, antibiotic resistance

## Abstract

Hypermutator lineages of *Pseudomonas aeruginosa* arise frequently during the years of airway infection experienced by patients with cystic fibrosis and bronchiectasis but are rare in the absence of chronic infection and structural lung disease. Since the onset of the COVID-19 pandemic, large numbers of patients have remained mechanically ventilated for extended periods of time. These patients are prone to acquire bacterial pathogens that persist for many weeks and have the opportunity to evolve within the pulmonary environment. However, little is known about what types of adaptations occur in these bacteria and whether these adaptations mimic those observed in chronic infections. We describe a COVID-19 patient with a secondary *P. aeruginosa* lung infection in whom the causative bacterium persisted for >50 days. Over the course of this infection, a hypermutator lineage of *P. aeruginosa* emerged and co-existed with a non-hypermutator lineage. Compared to the parental lineage, the hypermutator lineage evolved to be less cytotoxic and less virulent. Genomic analyses of the hypermutator lineage identified numerous mutations, including in the mismatch repair gene *mutL* and other genes frequently mutated in individuals with cystic fibrosis. Together, these findings demonstrate that hypermutator lineages can emerge when *P. aeruginosa* persists following acute infections such as ventilator-associated pneumonia and that these lineages have the potential to affect patient outcomes.

**IMPORTANCE:**

*Pseudomonas aeruginosa* may evolve to accumulate large numbers of mutations in the context of chronic infections such as those that occur in individuals with cystic fibrosis. However, these “hypermutator” lineages are rare following acute infections. Here, we describe a non-cystic fibrosis patient with COVID-19 pneumonia who remained mechanically ventilated for months. The patient became infected with a strain of *P. aeruginosa* that evolved to become a hypermutator. We demonstrate that hypermutation led to changes in cytotoxicity and virulence. These findings are important because they demonstrate that *P. aeruginosa* hypermutators can emerge following acute infections and that they have the potential to affect patient outcomes in this setting.

## INTRODUCTION

Many bacterial pathogens experience substantial stress as they transition from their natural reservoirs to the human body in the process of causing an infection. The immune system, intrinsic defenses, and altered nutrient availability together create an inhospitable environment to which the pathogen must adapt. Added to this are medically administered therapies such as antibiotics designed to eradicate the pathogen. To persist, the pathogen must adapt by the action of pre-programmed sensing systems or by the acquisition of mutations. Adaptation to the human host has been most thoroughly studied in the context of chronic infections lasting for years. The role of adaptation in acute or subacute infections remains less clear.

Long-term respiratory infections with the bacterium *Pseudomonas aeruginosa* in individuals with cystic fibrosis (CF) have served as a model for the study of bacterial adaptation. These patients tend to remain infected with a single lineage of *P. aeruginosa* for decades. Well-characterized adaptations in this context include the emergence of a mucoid colony morphology, antibiotic resistance, loss of type III secretion, loss of motility, quorum-sensing defects, and auxotrophy (1). Interestingly, emergence of these traits appears to be accelerated by the presence of a “hypermutator” phenotype in which an increased spontaneous mutation rate occurs following defects in DNA repair genes. Such hypermutator strains become fixed in populations exposed to new conditions in which the rapid emergence of mutations provides an adaptive advantage (2). In *P. aeruginosa*, hypermutability is most often a consequence of disruptions of mismatch repair genes, such as *mutS*, *mutL*, and *uvrD* (3).

Two especially well-studied phenotypes associated with hypermutator lineages of *P. aeruginosa* in CF are the emergence of antibiotic resistance and altered pathogenicity. In general, hypermutator strains have a higher prevalence of resistance to antibiotics than non-mutators (4–6). Indeed, hypermutator strains acquire resistance to antibiotics more quickly than non-mutator strains (6–8). Conversely, the same inactivation of the mismatch repair genes reduces *in vitro* fitness and attenuates virulence in acute infections. Hypermutator isolates recovered from CF patients demonstrated defects in prominent pathogenic systems such as type III secretion (9) and quorum sensing (10). Together, these observations indicate that the emergence of a hypermutator phenotype can have profound effects on antibiotic resistance and pathogenicity.

Hypermutator strains of *P. aeruginosa* are common in CF and other chronic infections associated with structural lung disease (4, 11–13). In contrast, these strains are relatively rare in acute infections such as ventilator-associated pneumonia (VAP) in the absence of pre-existing structural lung abnormalities (4, 14). This has led to speculation that hypermutators are selected against in acute infections (4). However, the COVID-19 epidemic has led to a large population of patients undergoing prolonged mechanical ventilation (15). These patients are at risk for repeated bouts of *P. aeruginosa* VAP and may harbor this bacterium for weeks. Whether these conditions allow for the emergence of hypermutators and whether such emergence has the potential to affect the treatment and outcome of pneumonia remains unclear.

Here, we present a non-CF patient with COVID-19 acute respiratory distress syndrome (ARDS) complicated by a protracted case of VAP during which *P. aeruginosa* developed a hypermutator phenotype. Over the course of the infection, a large number of mutations occurred in the *P. aeruginosa* lineage, including some that were associated with marked changes in antibiotic resistance and pathogenicity. This case demonstrates that hypermutators can evolve in the absence of chronic infections and have the potential to affect patient outcomes.

## MATERIALS AND METHODS

### Patient data and isolate collection

Clinical information was obtained from the hospital medical records. Clinical bacterial isolates were not available from the outside hospital to which the patient was initially admitted but were obtained from the Northwestern Memorial Hospital (NMH) microbiology laboratory during routine clinical care. In some cases, multiple isolates were obtained from a single bronchoalveolar lavage (BAL) procedure because of differing morphologies or sampling from different portions of the lung. Each isolate was archived at −80°C. For certain experiments, *P. aeruginosa* strains PA14, PA14Δ*exoU*, PA103, and PAO1 (16–18) were used as controls. This study was approved by the Northwestern University Institutional Review Board; informed consent was obtained.

### Whole-genome sequencing

Isolates were grown overnight in Luria Bertani (LB) broth with shaking at 37°C. DNA extraction was performed using Cell DNA Purification Kits (Promega Corporation, Madison, WI, USA) on a Maxwell 16 Instrument according to the manufacturer’s instructions. For short-read sequencing, libraries were prepared using Nextera XT Kits (Illumina, Inc., San Diego, CA, USA) and sequenced using an Illumina NextSeq (150-bp paired-end reads, high-output) platform. Sequences were trimmed using Trimmomatic v0.32 (19), and *de novo* assembly was performed with SPAdes v3.9.1. Contigs shorter than 200 bp or with a mean fold coverage of <5× per base were removed. For strain L00-a, additional long-read sequencing was performed using the MinION platform (Oxford Nanopore, UK), and hybrid assembly with short-read Illumina sequences was performed. Briefly, long-read sequencing libraries were prepared from unsheared genomic DNA using ligation sequencing kit SQK-LSK109 (Oxford Nanopore) and sequenced on the MinION platform using a FLO-MIN106 flow cell. Guppy v3.4.5 was used to base call reads with the R9.4.1 high-accuracy model and to perform read quality filtering based on *Q* scores, demultiplexing, and barcode trimming. Assembly of Nanopore reads was performed using the Trycycler v0.5.0 pipeline (20) as follows: raw nanopore reads were first filtered using Filtlong v0.2.1 (https://github.com/rrwick/Filtlong) to remove reads shorter than 1,000 bases and the 5% of reads with the lowest quality. Reads were then subsampled into 12 subsets using the Trycycler “subsample” function. Four read subsets were each assembled using (i) Flye v2.8.3 (21) or (ii) Raven v1.5.3 (22) or (iii) minimap2 v2.21, miniasm v0.3, and minipolish v0.1.3 via the “miniasm_and_minipolish.sh” script (https://github.com/rrwick/Minipolish/blob/main/miniasm_and_minipolish.sh). Clustering, reconciliation, circularization, multiple sequence alignment, and consensus sequence generation from the 12 assemblies were performed using Trycycler. The resulting consensus assembly was polished with Nanopore long reads and medaka v1.4.3 using the “r941_min_hac_g507” model. Illumina reads were aligned to the assembly using BWA v0.7.17 (23), and assembly errors were corrected using Pilon v1.23 (24) with a minimum depth setting of 0.1. Serial read alignment and Pilon correction were performed iteratively until no further assembly corrections were generated. Polypolish v0.4.3 (25) was used to further correct the assembly using the Illumina reads. Custom software (Pilon Tools v0.1; https://github.com/egonozer/pilon_tools) was used to identify, manually assess, and correct any residual homopolymer assembly errors.

### SNV identification and phylogenetic analysis

Illumina short reads from each isolate were aligned to the complete genome sequence of strain L00-a using BWA v0.7.15 (23). Single-nucleotide variants (SNVs) relative to this reference were identified using bcftools v1.9 with a haploid model and skipping of bases with quality lower than 25 or alignment quality less than 30. SNVs were further filtered as previously described (26) using the bcftools_filter software (https://github.com/egonozer/bcftools_filter) to remove variants with SNV quality scores less than 200, read consensuses less than 75%, read depths less than 5, read numbers in each direction less than 1, or locations within repetitive regions (as defined by BLAST alignment of the reference genome sequence against itself). The resulting multiple sequence alignments were filtered to include only variant positions with a defined base in each of the 22 isolates (i.e., the 100% core genome). A maximum likelihood phylogenetic tree was generated from the core genome alignment with IQ-TREE v1.6.1 using the ModelFinder function to estimate the best-fit nucleotide substitution model by means of the Bayesian information criterion (27, 28). Tree topology was assessed both with the Shimodaira-Hsegawa approximate likelihood ratio test and with the ultrafast bootstrap with 1,000 replicates each (29, 30).

### Rifampin resistance assays

The frequencies of mutations were quantified using assays to measure the emergence of resistance to rifampin, as described by Oliver et al. (11). Briefly, a bacterial colony was resuspended in 20 mL of Mueller-Hinton (MH) broth and grown at 37°C overnight. Bacterial cells were then collected by centrifugation at 2,500 × *g* for 5 min. Bacteria were resuspended in 1 mL of MH broth, and either 10- or 100-µL aliquots of serially diluted samples were plated onto MH agar plates with and without rifampin supplementation (300 µg/mL). Each isolate had initially demonstrated susceptibility to this concentration of rifampin. The plates were incubated at 37°C for 24–48 hr, and colonies were counted. The frequency of rifampin mutations was determined by dividing the number of CFU on MHB supplemented with rifampin by the number of CFU on MHB alone. A strain was designated a hypermutator if the number of colonies observed on rifampicin-supplemented agar was at least 20-fold higher than the number of colonies observed for the reference strain PAO1 (11). The number of replicates performed for each experiment is indicated in the figure legend showing these results.

### Antibiotic susceptibility assays

Samples from cryopreserved stocks were streaked onto LB agar plates and grown overnight at 37°C. Bacterial colonies were resuspended in sterile water and adjusted to an optical density at 600 nm (OD_600_) of 0.08–0.1. A total of 30 µL of the bacterial suspensions was then diluted in 11 mL of MH broth, and 50 µL of this suspension was distributed into pre-manufactured Sensititre GNX3F plates (Thermo Fisher Scientific, UK). The plates were incubated for 20 h and were manually read. All isolates grew in the control wells lacking antibiotics. Clinical and Laboratory Standards Institute 2021 breakpoints for *P. aeruginosa* were used to define isolates as susceptible, intermediately susceptible, or resistant. For each isolate, assays were performed in duplicate. If duplicate assays differed by one dilution, the value of the higher dilution was reported. If assay results differed by more than one dilution, the assay was performed a third time, and the majority result was reported.

### Biofilm assays

A single bacterial colony was re-suspended in 5 mL of LB broth and grown overnight at 37°C at 250 rpm. The following day, the culture was diluted 1:100 in M9 minimal media with MgSO_4_ and CaCl_2_ (1.2% Na_2_HPO_4_ 7 H_2_O, 0.6% KH_2_PO_4_, 0.1% NaCl, 0.2% NH_4_Cl, 0.4% glucose, 2 mM MgSO_4_, and 0.1 mM CaCl_2_) in a 96-well plate and incubated in a 37°C static incubator for 16 h. After incubation, the liquid was removed, and the plate was air-dried for 30 min. Water containing 0.1% crystal violet was added to each well and incubated for 10 min at room temperature. The crystal violet solution was removed, and the wells were washed three times with water to remove unbound dye and bacteria. The plate was air-dried for at least 45 min. Then, 30% acetic acid in water was added to each well and incubated for 30 min at room temperature to solubilize the crystal violet. Absorbance at 530 nm was read using a SpectraMax iD3 Multi-Mode Microplate Reader. The number of replicates performed for each experiment is indicated in the figure legend showing these results.

### Bacterial growth assays

A single bacterial colony was inoculated into 5 mL of LB broth and grown overnight at 37°C at 250 rpm. The following morning, the bacteria was sub-cultured 1:100 in fresh LB for 3 h and then adjusted to an OD_600_ of 0.1 in LB broth. Volumes of 200 µL/well were added to a 96-well clear, flat-bottom plate (Corning, Corning, NY, USA). The plate was incubated at 37°C with shaking, and bacterial growth was quantified using a SpectraMax iD3 Multi-Mode Microplate Reader to measure OD_600_ every 30 min over 24 h. The R package Growthcurver (31) was then used to determine the growth rate from each growth curve. The number of replicates performed for each experiment is indicated in the figure legend showing these results.

### Cytotoxicity assays

Cytotoxicity (mammalian cell death) was assessed by the use of quantitative lactate dehydrogenase (LDH) release assays (CyQUANT LDH Cytotoxicity Assay, Invitrogen-Thermo Fisher). Bacteria were inoculated into 5 mL of LB broth and grown overnight at 37°C with shaking. The bacteria were pelleted by centrifugation and resuspended in phosphate-buffered saline (PBS) to an appropriate concentration. Approximately 20,000 A549 cells were seeded into 96-well tissue culture-treated plates with Dulbecco’s modified Eagle medium containing 10% fetal bovine serum. A549 cells were grown for 22 h, after which the culture medium was replaced with RPMI 1640 medium without phenol red. A total of 10 µL of bacterial suspension (~400,000 CFU) was added to each well to achieve an MOI of ~10. The negative control was RPMI 1640 medium without phenol red containing PBS in place of bacteria. The positive control was RPMI 1640 medium without phenol red containing 0.05% Triton X-100, which lysed all cells. After 3 and 8 h, the plate was spun down at 500 × *g* for 5 min, and LDH released into the medium was measured per the manufacturer’s instructions. The number of replicates performed for each experiment is indicated in the figure legend showing these results.

### Immunoblot analyses

For detection of ExoU secretion, isolates were grown in LB broth overnight at 37°C at 250 rpm. The bacteria were sub-cultured in LB broth supplemented with MgCl_2_ (20 mM) and EGTA (5 mM) for 3 h at 37°C at 250 rpm. Calcium chelation induces type III secretion of ExoU. Bacterial supernatants were collected by centrifugation at 2,500 × *g* for 10 min at room temperature. The supernatants were passed through 0.22-µm filters and incubated with trichloroacetic acid (20%) overnight at 4°C with slow shaking. The precipitated proteins were collected by centrifugation at 8,650 × *g* for 45 min at 4°C, washed two times with acetone, and then re-suspended in Laemmli Sample Buffer (Bio-Rad, Hercules, CA, USA). The samples were heated to 80°C for 10 min, and 20 µL of each sample was electrophoresed through a 4%–15% precast polyacrylamide gel (Bio-Rad, Hercules, CA, USA). Proteins were then electrotransferred onto a nitrocellulose membrane (Schleicher and Schuell). The membrane was blocked with 5% milk powder (Boston BioProducts, Ashland, MA, USA) in 1× Tris-buffered saline (Bio-Rad, Hercules, CA, USA) with 0.5% Tween-20 (TBS-T; National Diagnostics, Atlanta, GA, USA) for 1 h at room temperature and then incubated with a 1:2,000 dilution of polyclonal anti-ExoU antibodies overnight at 4°C on a slow rocker (32). Polyclonal ExoU antiserum was prepared as described elsewhere (33). The membrane was washed three times with 1× TBS-T at room temperature and then exposed to a goat anti-rabbit secondary antibody (LI-COR Biosciences, Lincoln, NE, USA) at a 1:5,000 dilution in 20 mL of 5% milk buffer in 1× TBS-T for 1 h at room temperature. Membranes were incubated in TBS-T and visualized using a LI-COR Biosciences Odyssey Fc Imaging System.

### Mouse model of acute pneumonia

Bacteria were tested in a mouse model of acute pneumonia, as previously described (34). Briefly, bacteria were sub-cultured in LB medium at 37°C in a shaking incubator, collected by centrifugation at 2,500 × *g* for 10 min, washed once in PBS, and re-suspended in PBS. Appropriate bacterial concentrations were estimated by diluting with PBS and measuring the OD_600_. Estimates were verified by plating serial dilutions and counting CFU. Six- to eight-week-old BALB/C mice purchased from Envigo were anesthetized with intraperitoneal ketamine (100 mg/kg) and xylazine (20 mg/kg). A total of 50 µL of the bacterial suspension was inoculated intranasally. Mice were monitored over the subsequent 7 days and euthanized and scored as dead when they met pre-determined criteria: loss of >20% body weight from baseline, loss of >10% body weight from previous measurement, body condition score of <2 (i.e., emaciated), hunched posture with minimal activity, or respiratory rates of <90 or >170/min. The 50% lethal dose (LD_50_) was calculated using the R package “drc” (35). All procedures were conducted in accordance with and approved by the Northwestern University Animal Care and Use Committee. The number of replicates performed for each experiment is indicated in the figure legend showing these results.

### Statistical analyses

All statistics were performed using R (v4.3.0) or Prism. The statistical test used to detect differences is indicated in the figure legend showing the results for each experiment. *P* values of ≤0.05 were considered significant.

## RESULTS

### Case presentation

**Fig 1:**
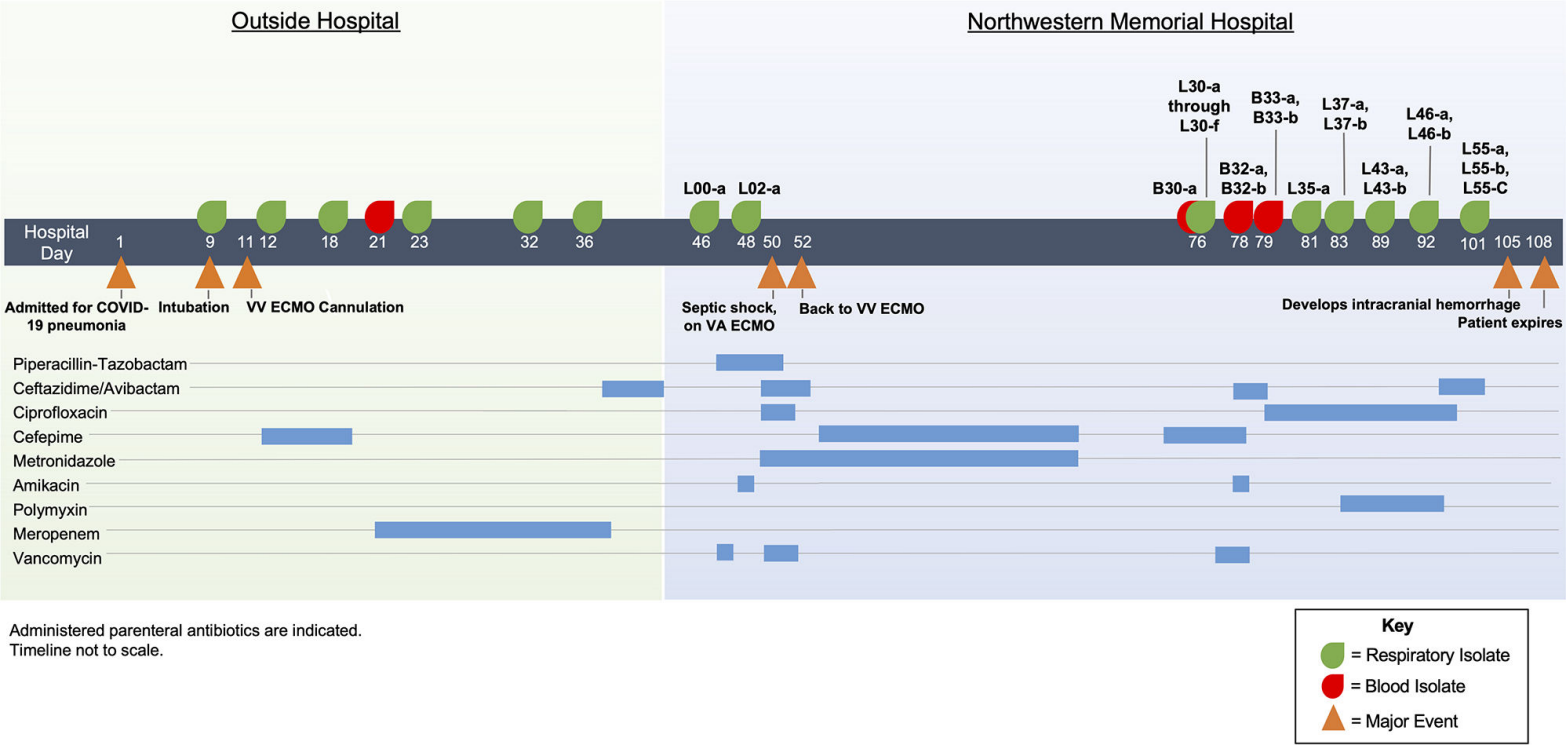
Clinical course of the patient. Events that occurred at the outside hospital are shaded in light green, and events that occurred at the NMH are shaded in light blue. Major medical events are indicated with orange triangles below the timeline. Cultures that grew *P. aeruginosa* are indicated above the timeline along with the names of isolates used in the study.

The patient, a previously healthy person in his 50s with no significant prior past medical history, was admitted to an outside hospital with COVID-19 pneumonia (Fig. 1). They required mechanical ventilation on hospital day 9. They subsequently developed ARDS and, on hospital day 11, required veno-venous extracorporeal membrane oxygenation (VV ECMO) support. Respiratory cultures obtained by bronchoscopy on hospital days 9 and 12 grew *P. aeruginosa*/*Klebsiella aerogenes* and *P. aeruginosa*, respectively, and a 7-day course of cefepime was started. On hospital day 18, another bronchoscopy was performed, and BAL fluid again grew *P. aeruginosa*. On hospital day 21, blood cultures grew *P. aeruginosa*, and vancomycin and meropenem were started; the vancomycin was discontinued after 1 day, and the meropenem was continued for 18 days. On hospital days 23 and 32, BAL fluid grew *P. aeruginosa* and *Serratia marcescens*, and on hospital day 36, BAL fluid grew *P. aeruginosa*. On hospital day 40, the patient was transitioned from meropenem to ceftazidime-avibactam, which was continued for 6 days. The patient was transferred to the NMH on hospital day 46 for consideration for lung transplantation in the setting of COVID-19 ARDS. On that day and 2 days later, BAL fluid was obtained that subsequently grew *P. aeruginosa*. Computed tomography imaging of the lungs was notable for a right middle lobe fluid collection concerning for an abscess. Piperacillin-tazobactam was started, and the subsequent course was complicated by a tension hemopneumothorax secondary to traumatic chest tube placement. On hospital day 50, the patient developed septic shock with worsening lung function. Antibiotics were changed to ceftazidime-avibactam plus ciprofloxacin, and veno-arterial ECMO was started. The patient’s condition stabilized, and they were transitioned to VV ECMO. Two days later, antibiotics were changed to cefepime, metronidazole, and inhaled amikacin, which were continued until the completion of a 2-week course. On hospital day 76, the patient became hemodynamically unstable, and blood cultures and BAL fluid again grew *P. aeruginosa*. Cefepime was started but was changed to ceftazidime-avibactam 2 days later. Blood cultures drawn on hospital days 78 and 79 remained positive for *P. aeruginosa*, and on hospital day 80, antibiotics were transitioned to ciprofloxacin plus polymyxin B based on susceptibility results. On hospital days 81, 83, 89, 92, and 101, BAL fluid samples were obtained that all subsequently grew *P. aeruginosa*. Unfortunately, the patient was ultimately determined to be a poor transplantation candidate, and withdrawal of supportive measures ensued. The patient succumbed to his illness on hospital day 108.

### Phylogenetic relationships of *P. aeruginosa* isolates

**Fig 2:**
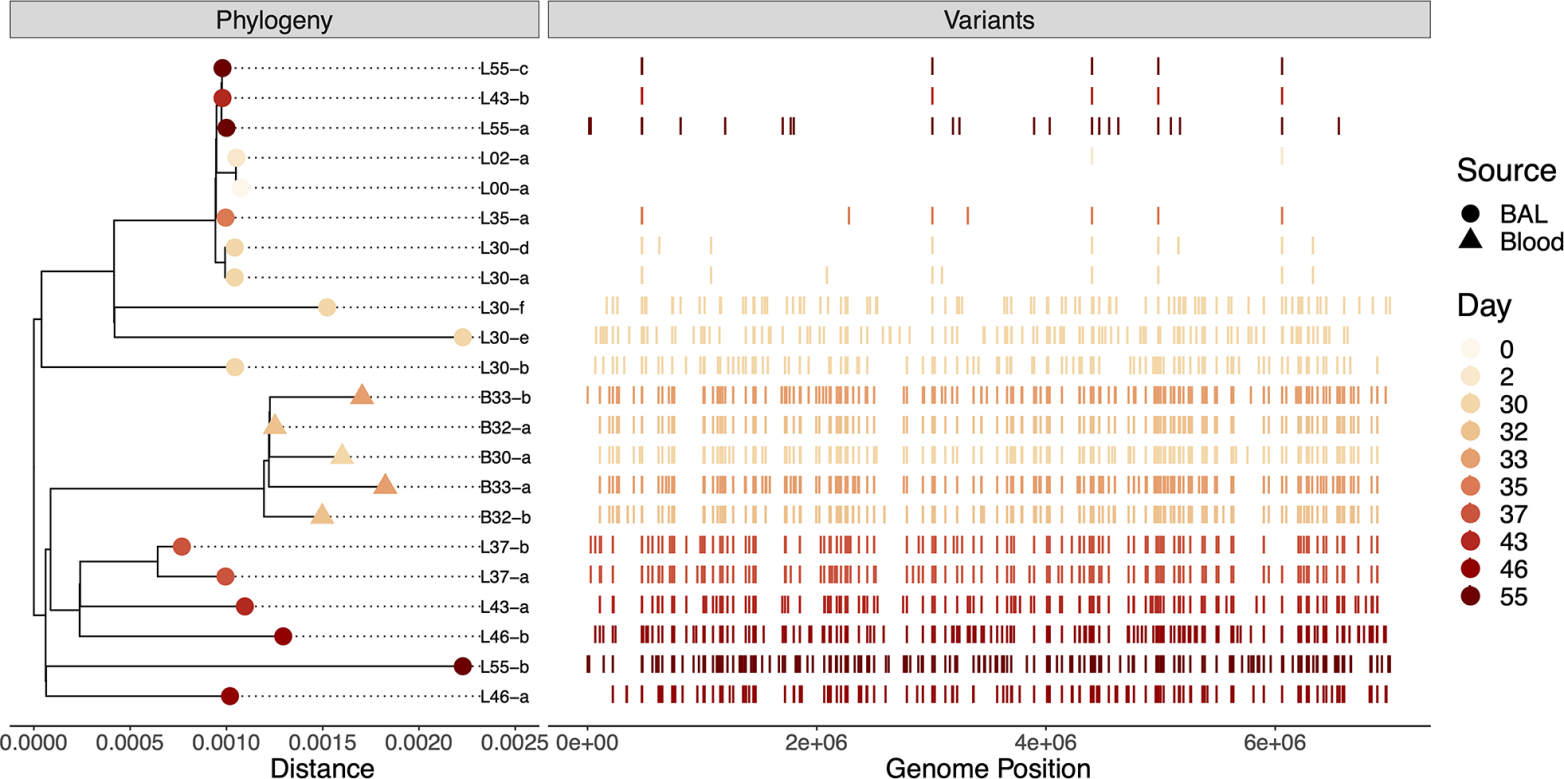
Phylogeny and SNVs of *P. aeruginosa* isolates. On the left, a maximum likelihood core genome phylogenetic tree is shown. On the right, the locations of SNVs in each isolate relative to the L00-a genome sequence are indicated.

A total of 22 *P*. *aeruginosa* isolates from the patient were available for analysis (Fig. 1). The complete genome sequence of the first available *P. aeruginosa* isolate (L00-a from hospital day 46) was obtained using the hybrid assembly of reads from both Nanopore and Illumina platforms. The remaining 21 isolates were sequenced using the Illumina platform (Table S1) and were aligned to the L00-a genome to identify SNVs that evolved during the infection (Table S2). Phylogenetic analysis of the 22 sequences demonstrated that all were of the same lineage (i.e., all isolates were descendants of the same initial infecting bacterium) (Fig. 2). However, two distinct patterns were readily apparent. Many of the isolates, including the isolate collected 2 days after L00-a, possessed relatively few (5–21) SNVs and were nearly identical to L00-a (Fig. 2). Isolates from this group were cultured throughout the patient’s hospitalization. However, beginning on hospital day 76, many isolates were obtained that had acquired relatively large numbers of SNVs (>100). Isolates such as these were also present throughout the remaining course of infection, co-existed with the first lineage, and were cultured from both respiratory and blood samples.

### Hypermutator analyses

**Fig 3:**
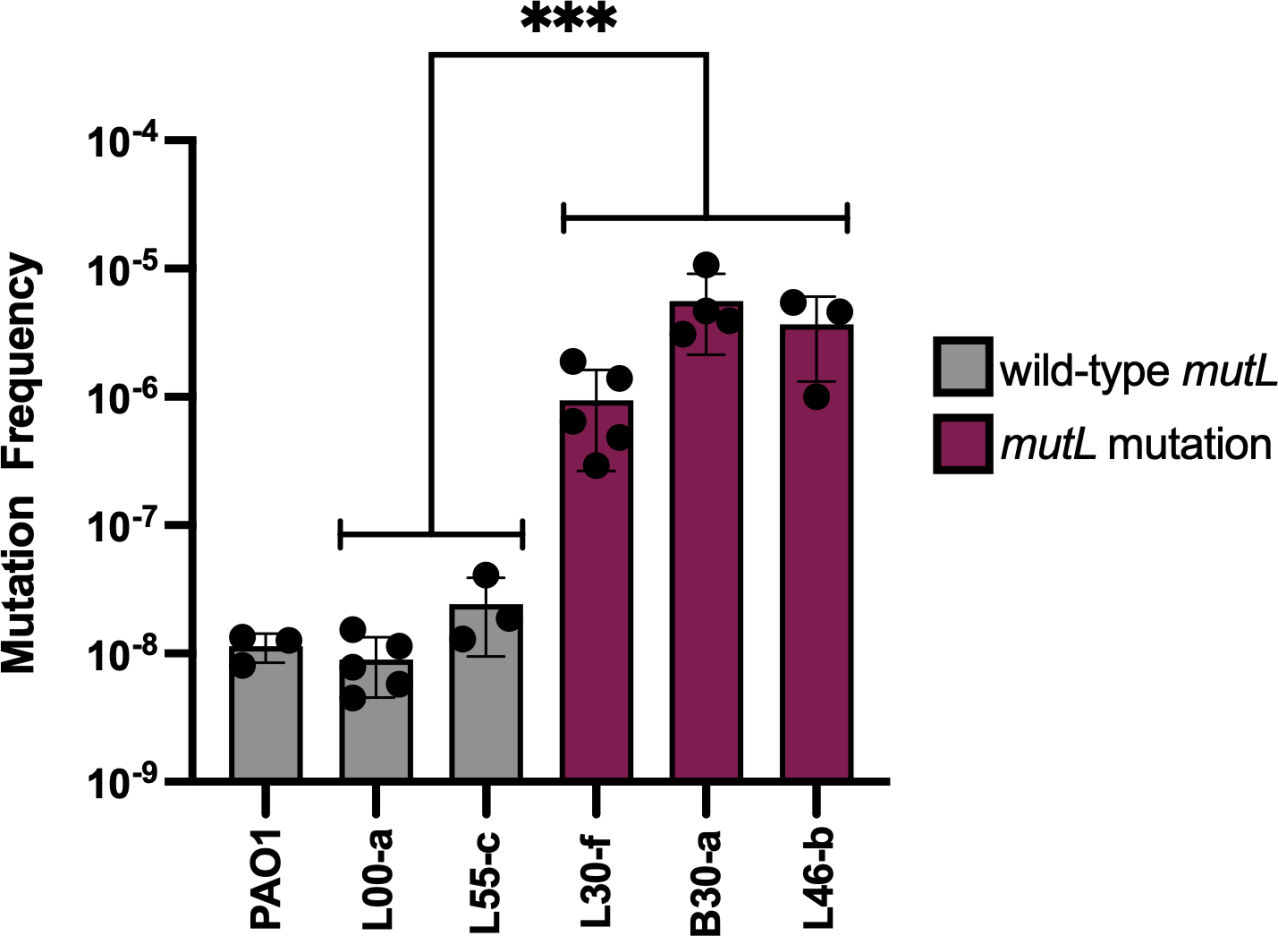
Emergence of resistance to rifampin in representative *P. aeruginosa* isolates with or without mutations in *mutL*. PAO1 is a reference non-hypermutator *P. aeruginosa* strain. Bars represent means of three to five biological replicates. Error bars represent standard deviations. The mean value of the non-hypermutator isolates was statistically different from the mean value of the hypermutator isolates (Mann-Whitney *U*, *P* = 0.00021).

The subpopulation of *P. aeruginosa* isolates containing numerous mutations that evolved over the relatively short time of the patient’s hospitalization suggested a hypermutator strain (10). Indeed, a SNV yielding a non-synonymous single amino acid substitution in the mismatch repair gene *mutL* was noted in each of the putative hypermutator isolates but in none of the other isolates (Table S2). To further explore the significance of this mutation, we performed assays to measure the emergence of resistance to rifampin, which are commonly used to identify hypermutators (10). Since resistance to rifampin requires a single mutation, the spontaneous emergence of rifampin resistance can be used as a phenotypic surrogate to measure the frequency of mutation (36). We selected three representative isolates with the *mutL* SNV and two isolates without the *mutL* SNV. We also included the non-hypermutator reference strain PAO1. Broth cultures of isolates were grown in the absence of rifampin and then plated on agar with or without rifampin supplementation to quantify the rate at which rifampin resistance emerged in strains with wild-type or mutated *mutL*. The three isolates containing *mutL* mutations evolved rifampin resistance at approximately 30- to 550-fold greater frequency than the isolates containing intact *mutL*, including the reference strain PAO1 (Fig. 3). These findings confirm that a *P. aeruginosa* hypermutator lineage evolved during the course of the patient’s infection.

### Antibiotic resistance

**Fig 4:**
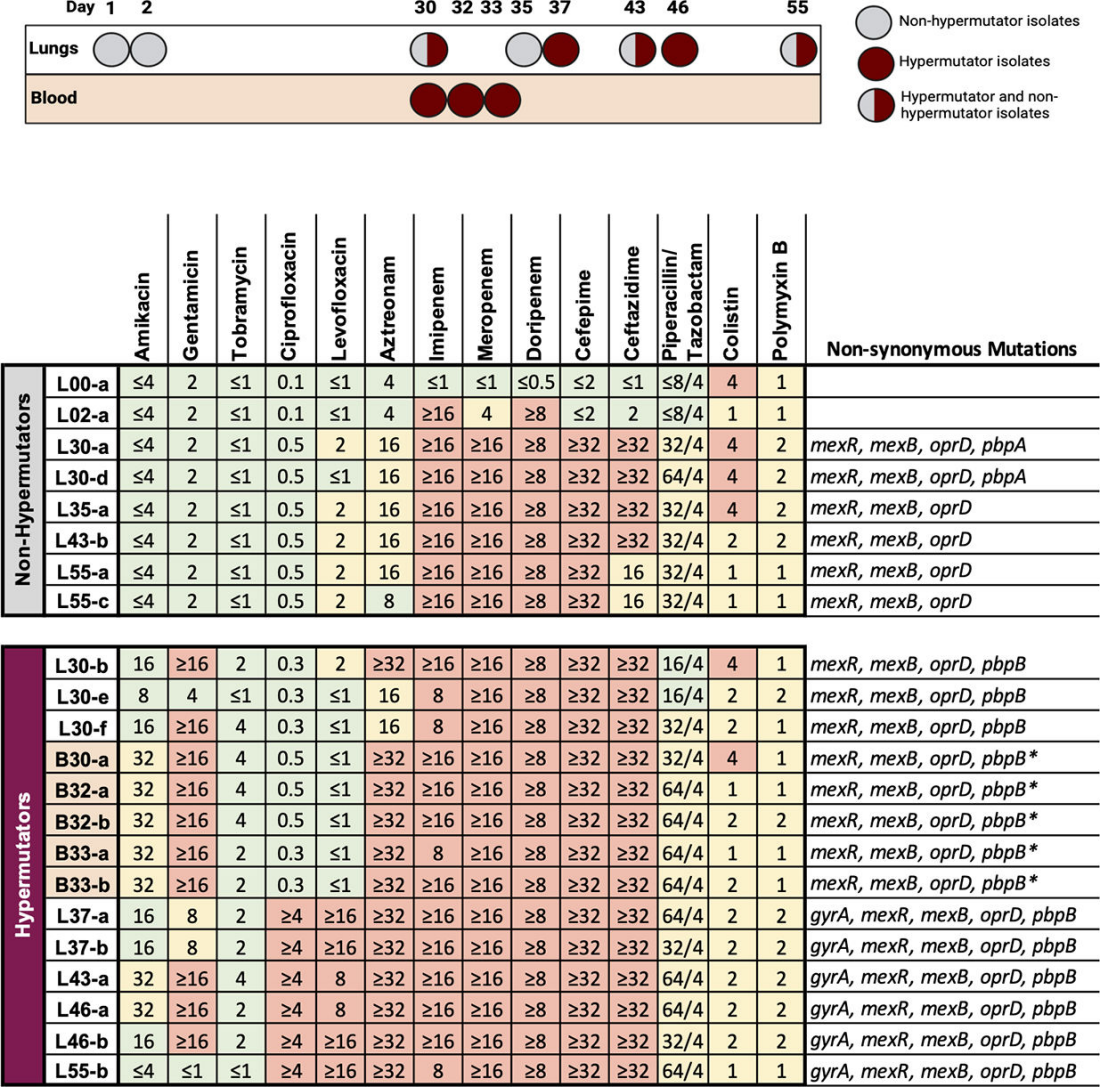
Culture timeline and antibiotic MICs of *P. aeruginosa* isolates. Susceptible MICs are shown in green, intermediately susceptible MICs in yellow, and resistant MICs in red. Known antibiotic resistance genes containing non-synonymous mutations in the indicated isolates are shown to the right of the table. For reference, a timeline showing when each isolate was cultured is shown above the table (created with BioRender.com). The white background shading indicates lung isolates, and the orange background shading indicates blood isolates. Each MIC assay was performed a minimum of two times. * indicates the presence of multiple non-synonymous mutations.

One of the fitness pressures that may select for the emergence of hypermutator strains is the inhibitory activity of antibiotics. Hypermutator strains acquire resistance to antibiotics more quickly than conventional strains, which may allow them to better persist in the face of antimicrobial therapy (4–6). As mentioned, this patient was heavily exposed to antipseudomonal antibiotics, including piperacillin-tazobactam, ceftazidime-avibactam, ciprofloxacin, cefepime, polymyxin B, and inhaled amikacin (Fig. 1). We therefore examined the antibiotic susceptibilities of the isolates using broth dilution assays. *P. aeruginosa* isolates cultured at the outside hospital already showed resistance to aztreonam, cephalosporins, and carbapenems (Table S3). Unfortunately, these isolates were not available for further analysis. Following transfer to the NMH, initial cultures grew *P. aeruginosa* (L00-a) that was susceptible to each of the tested antibiotics (Fig. 4). However, antibiotic resistance subsequently emerged in both hypermutator and non-hypermutator lineages. An isolate cultured only 2 days later (L02-a) had acquired resistance to imipenem and doripenem and intermediate susceptibility to meropenem. Non-hypermutator isolates collected on or after hospital day 76 (day 30 at the NMH) were largely resistant to carbapenems, cefepime, and ceftazidime, while hypermutator isolates that co-existed with these non-hypermutator isolates were resistant to these antibiotics plus gentamicin and aztreonam (Fig. 4). Beginning on hospital day 83 (day 37 at the NMH), hypermutator isolates were resistant to ciprofloxacin and levofloxacin. Although the non-hypermutator lineage presumably predated the hypermutator lineage and therefore was exposed to antibiotics for a longer time, hypermutator isolates were susceptible to fewer antibiotics than the non-hypermutator isolates (1 vs. 4 of the 14 tested antibiotics). However, the difference in antibiotic non-susceptibility measured by minimum inhibitory concentration (MIC)_90_ of hypermutator vs. non-hypermutator isolates was not statistically significant (chi-squared test; *P* = 0.14). These findings demonstrate that hypermutators can accumulate antibiotic resistance but fail to confirm that this occurs more rapidly than in non-hypermutators.

An analysis of the genomes of the *P. aeruginosa* isolates provided mechanistic explanations for some of the phenotypic antimicrobial resistance patterns. In both hypermutator and non-hypermutator lineages, intermediate susceptibility to aztreonam and resistance to meropenem, cefepime, and ceftazidime were associated with three changes that were apparent simultaneously in hospital day 76 (day 30 at the NMH) isolates: (i) glycine-to-tryptophan substitution at amino acid 101 of MexR, (ii) an arginine-to-cysteine substitution at amino acid 620 of MexB, and (iii) a substitution causing a premature stop codon in the gene encoding OprD (Fig. 4). Since these changes occurred in both lineages, they likely occurred prior to the emergence of the hypermutator lineage. MexR is a repressor of the MexAB-OprM efflux pump, and its disruption could lead to overexpression of MexAB-OprM, which has been linked to beta-lactam resistance (37). Likewise, the substitution in MexB has the potential to enhance the efflux of beta-lactams. Disruption of the OprD outer membrane porin contributes to high-level resistance to carbapenems (37). The hospital day 76 hypermutator isolates (but not the non-hypermutator isolates) also contained an arginine-to-histidine substitution at residue 504 of penicillin-binding protein 3 (encoded by *pbpB*), which may be responsible for the elevated MICs to aztreonam observed in this lineage (37). On hospital day 83 (hospital day 37 at the NMH), the hypermutator lineage acquired a threonine-to-isoleucine substitution in subunit A of DNA gyrase, which was associated with the development of resistance to ciprofloxacin and levofloxacin. These findings demonstrate how increased numbers of mutations at the genomic level may lead to phenotypic antibiotic resistance in hypermutator strains.

### Biofilm formation

**Fig 5:**
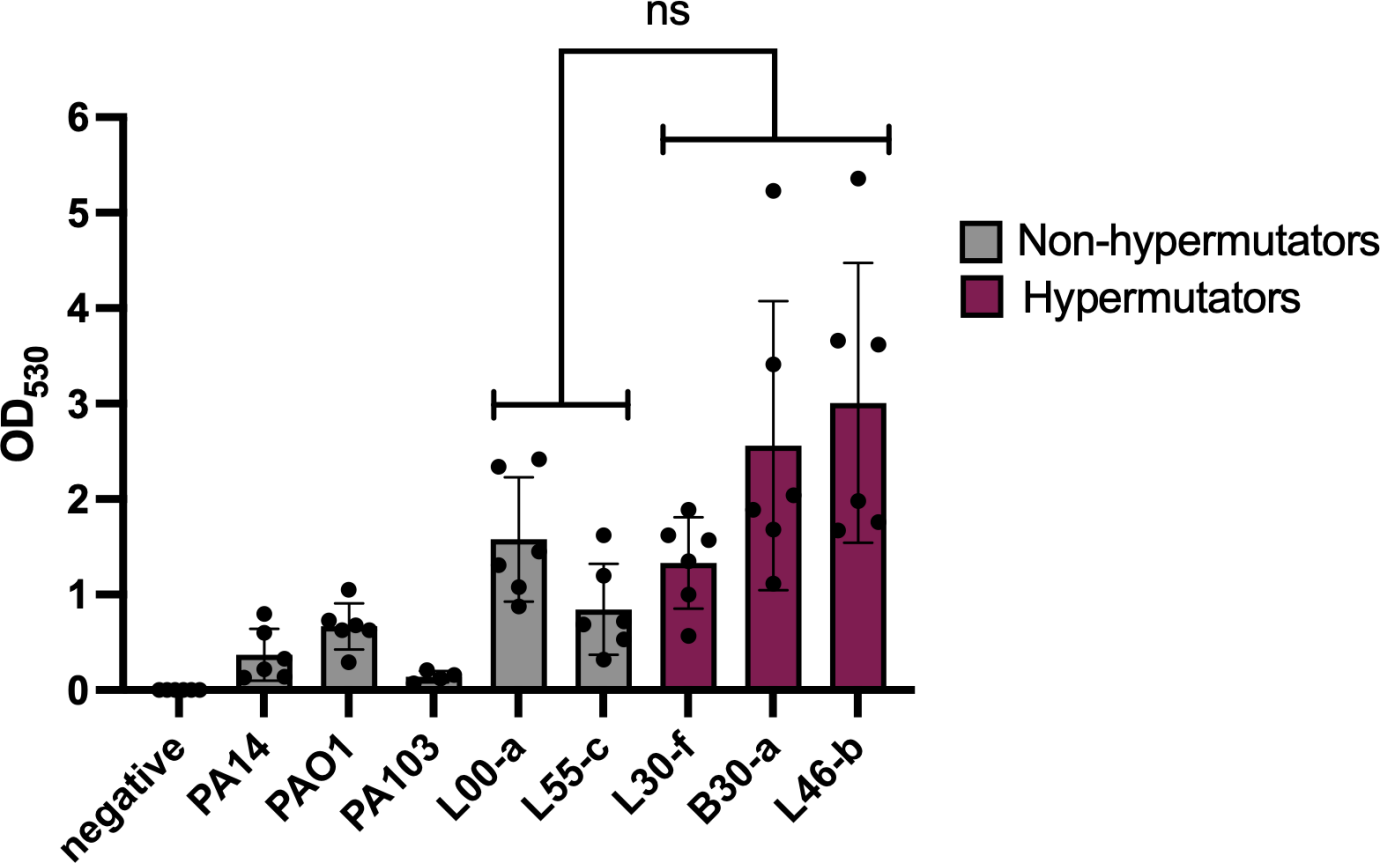
Biofilm formation by representative *P. aeruginosa* isolates. Bacteria were grown for 16 h, after which crystal violet was applied and biofilm mass was quantified. PA14, PAO1, and PA103 are non-hypermutator *P. aeruginosa* reference strains but were not included in the statistical analyses. Each dot represents a biological replicate (*n* = 6 except for PA103, which has n = 4); each biological replicate consists of three technical replicates. Bars represent means, and error bars represent standard deviations. “Negative” indicates no added bacteria. There was no statistical difference between the means of hypermutator and non-hypermutator isolates (*t*-test, *P* = 0.22). Post hoc analysis using paired *t*-test showed significant differences between L30-f/L46-b (*P* = 0.024), B30-a/L55-c (*P* = 0.024), and L46-b/L55-c (*P* = 0.0064).

We measured biofilm formation, as increased robustness of these bacterial communities may confer enhanced resistance to antibiotics and play a role in persistence. We performed crystal violet biofilm assays on three representative hypermutator isolates and two non-hypermutator isolates. We also tested three laboratory strains of *P. aeruginosa*. Although the amount of biofilm formation between non-hypermutators and hypermutators was not significant, two hypermutator isolates showed a trend toward more robust biofilm formation than the non-hypermutator isolates (Fig. 5). These findings did not confirm that hypermutator lineages have the potential to enhance biofilm formation.

### Growth rate

**Fig 6:**
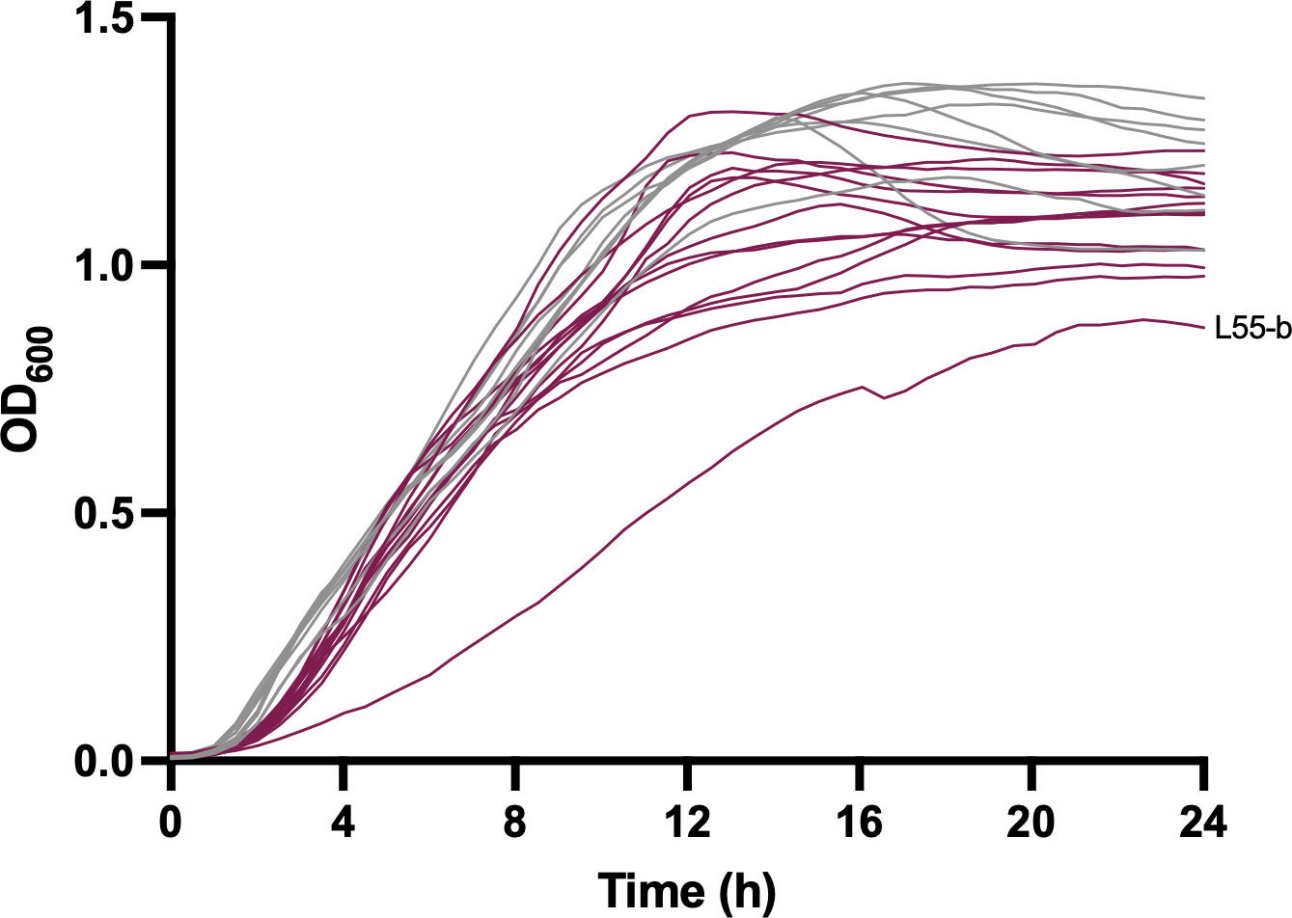
Growth curves of *P. aeruginosa* isolates. Each *P. aeruginosa* isolate was grown in LB medium. The growth rate was then determined for each isolate using Growthcurver. There is no significant difference between the growth rates of hypermutators and non-hypermutators (Student’s *t*-test, *P* = 0.25). As a post hoc analysis, we compared the growth rate of L55-b, a hypermutator isolate, to the rest of the hypermutators and found that L55-b did have a statistically significant slower growth rate compared to the other hypermutators (analysis of variance with Bonferroni correction for a significance level of 0.025, *P* = 0.0016). Growth curves were performed as two biological replicates, and a representative replicate is shown. Each curve represents the mean of three technical replicates.

Hypermutator strains contain large numbers of mutations, so one would anticipate that their growth rates may be affected. We therefore measured the growth rates of the 22 isolates in rich (LB) medium. Growth rates differed substantially among the different isolates (Fig. 6), but there was no statistically significant difference in the average growth rate of hypermutator isolates compared to non-hypermutator isolates. L55-b, a hypermutator isolate, did grow significantly more slowly than the other hypermutator isolates. Upon closer inspection, 48 non-synonymous mutations were found to occur only in L55-b (Table S2). Several of these were in genes that may play a key role in metabolism or cell replication and therefore contribute to the slow-growing phenotype of this isolate. These include the genes encoding a putative acyl-CoA dehydrogenase, an ATP-dependent DNA helicase, a putative amino acid permease, the Holliday junction resolvase RuvC, and topoisomerase IV subunit B. These findings show that the hypermutation phenotype has the potential to affect growth rate.

### Cytotoxicity

**Fig 7:**
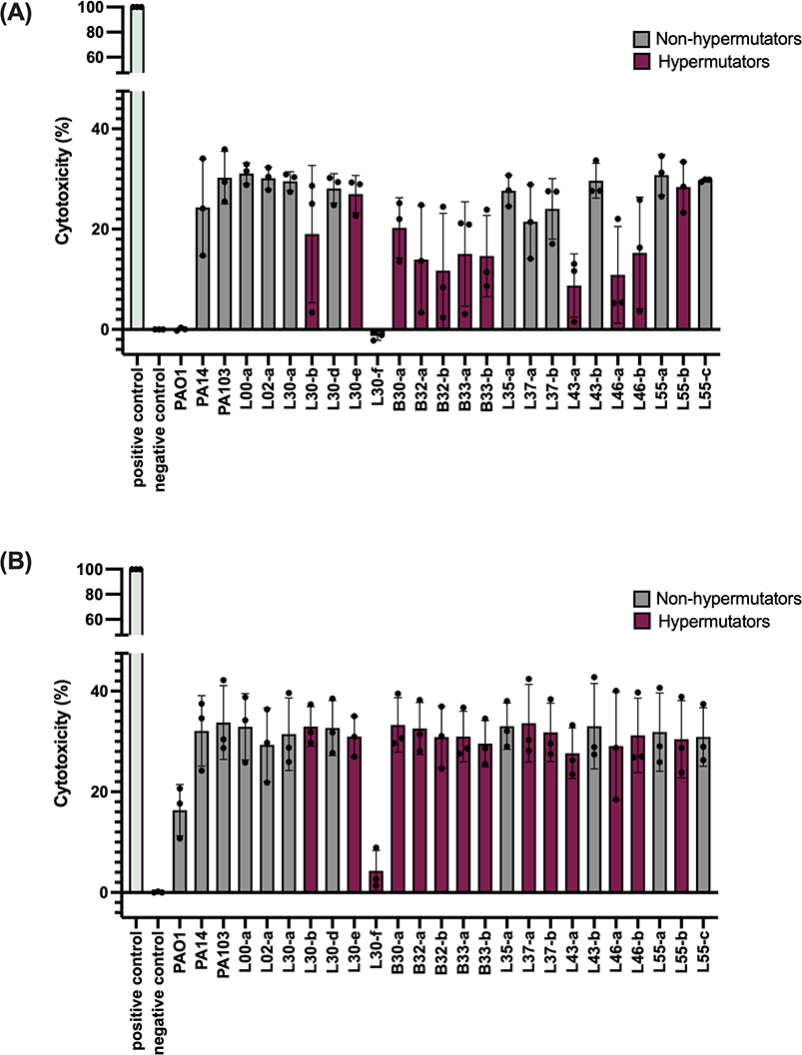
Cytotoxicity of *P. aeruginosa* isolates. Bacteria were co-incubated with A549 cells at a multiplicity of infection (MOI) of ~10 for (**A**) 3 and (**B**) 8 hr. PAO1, PA14, and PA103 are non-hypermutator *P. aeruginosa* reference strains. “Positive control” is lysis of all cells by triton detergent; “negative control” is the absence of added bacteria. Each dot represents a biological replicate (*n* = 3) consisting of three technical replicates. Bars represent means, and error bars represent standard deviations. As a group, the hypermutator isolates were less cytotoxic than the non-hypermutator isolates at 3 h (two-tailed *t*-test, *P* = 0.0001), although by 8 h, this difference was no longer apparent (*P* = 0.33).

Because hypermutator strains have been linked to changes in pathogenicity (38), we measured the ability of *P. aeruginosa* to kill human cells under cell culture conditions. We incubated the hypermutator and non-hypermutator isolates, as well as laboratory reference strains PAO1, PA14, and PA103, with A549 pulmonary epithelial-like cells for 3 and 8 h. As previously reported, PAO1 caused low levels of cytotoxicity, whereas PA14 and PA103 caused relatively high levels of cytotoxicity (39, 40) (Fig. 7). As a group, the hypermutator isolates were less cytotoxic than the non-hypermutator isolates at 3 h of co-incubation, although by 8 h, this difference was no longer apparent. An exception was the hypermutator isolate L30-f, which caused little cytotoxicity even after 8 h of co-incubation. These findings indicate that the hypermutator phenotype has the potential to attenuate *P. aeruginosa* cytotoxicity.

### Type III secretion phenotype

**Fig 8:**
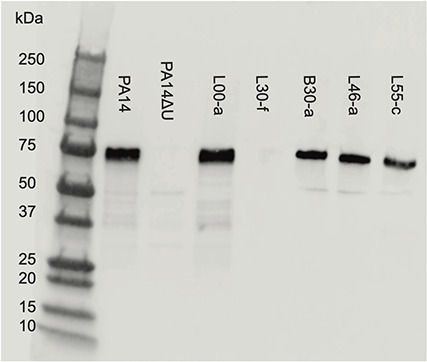
ExoU secretion by representative *P. aeruginosa* isolates. Bacterial culture supernatants were precipitated, partially purified, concentrated, and tested for the presence of ExoU using polyclonal anti-ExoU antibodies. PA14 is an ExoU-secreting reference strain, and PA14ΔU is the same strain containing a disruption of the *exoU* gene. Sizes of the molecular weight markers are indicated. Assays were performed in triplicate on all isolates. Representative isolates are shown.

The type III secretion system is a major virulence factor of *P. aeruginosa* and impacts its cytotoxicity (41). Strains that secrete the type III secretion effector protein ExoU, a phospholipase that degrades cell membranes, tend to be highly cytotoxic, and strains that do not secrete ExoU tend to cause lower levels of cytotoxicity (34). We examined the genome sequence of the *P. aeruginosa* lineage causing infection in our patient and found that it contained the *exoU* gene. We performed immunoblot analyses on all isolates to determine whether they indeed secreted ExoU. We also tested two control strains, PA14 and PA14Δ*exoU*, the latter of which contains a disrupted *exoU* gene. Bacteria were grown in a liquid medium under conditions that induced type III secretion. Culture supernatants were collected and partially purified. Proteins were separated by gel electrophoresis, transferred to membranes, and incubated with polyclonal anti-ExoU antibodies. As expected, PA14 secreted ExoU, whereas PA14Δ*exoU* did not (Fig. 8). Twenty-one of the 22 patient isolates secreted ExoU. Results from representative isolates are shown in Fig. 8. The sole exception was L30-f, a hypermutator isolate. These findings demonstrate that defects in type III secretion can evolve in hypermutator lineages, although it may be a relatively rare occurrence. Furthermore, these findings explain why L30-f caused little cytotoxicity when co-incubated with A549 cells (Fig. 7).

### Virulence in a mouse model

**Fig 9:**
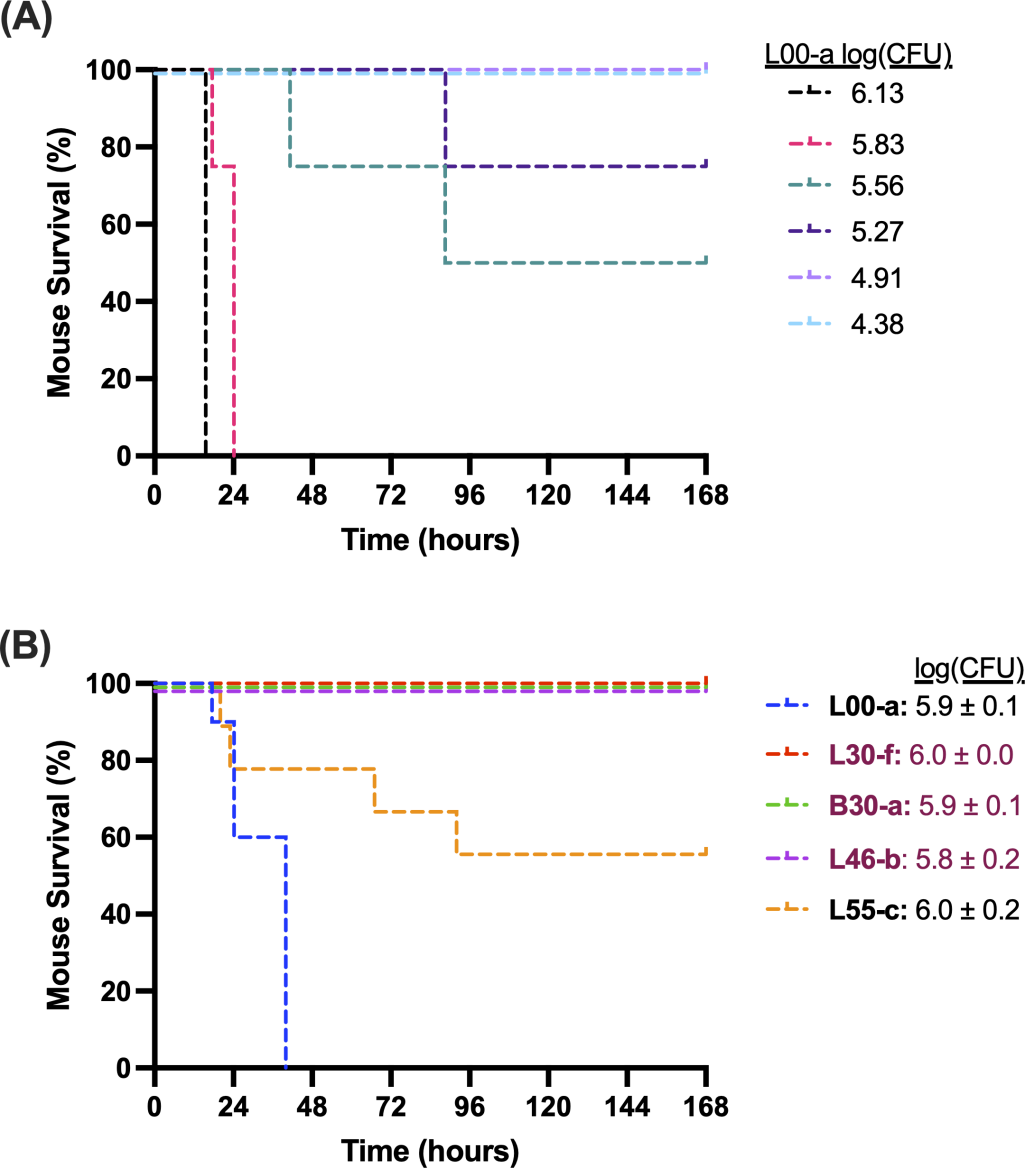
Virulence of representative *P. aeruginosa* isolates in a mouse model of acute pneumonia. (**A**) Estimation of the LD_50_ values of isolate L00-a. Inoculum sizes are shown to the right. A total of three to seven mice were used for each dose. (**B**) Comparison of survival of mice infected with different isolates. For each isolate, an inoculum size of ~10^6^ CFU was estimated by optical density. Actual inoculum sizes were then determined by plating and colony enumeration and are shown to the right. Each experiment (*n* = 5) was repeated on two separate days, and results combined for a total of 10 mice per isolate, except for L55-c, for which only nine mice were available because one mouse did not survive the anesthetic and inoculation process. The L00-a results shown in panel **A** for the dose of 10^5.83^ CFU are also included in panel **B**. As a group, the hypermutator isolates were significantly less virulent than the non-hypermutator isolates (log rank test; *P* = 6.0 × 10^−9^).

We next measured the virulence of several representative isolates using a mouse model of pneumonia. In this model, most previously tested ExoU^+^ strains of *P. aeruginosa* have had 50% pre-lethal dose (LD_50_) values of ~10^7^ CFU (~10,000,000 CFU) (34). In contrast, the initial isolate from our patient, L00-a, had an LD_50_ value of 10^5.6^ CFU (~400,000 CFU) (Fig. 9A). Based on this result, L00-a is one of the most virulent strains of *P. aeruginosa* we have tested in this model system (34). We next inoculated mice with a slightly larger dose (~10^6.0^ CFU) of the following isolates: non-hypermutator isolates L00-a and L55-c and hypermutator isolates L30-f, B30-a, and L46-b. As expected, all mice inoculated with L00-a developed pre-lethal illness within the first 48 h (Fig. 9B). Interestingly, all mice inoculated with each of the three hypermutator isolates survived. Even the mice inoculated with the non-hypermutator isolate L55-c, which had been cultured late in the patient’s infection, showed increased survival relative to L00-a. These findings indicate that both the hypermutator and non-hypermutator lineages evolved to become less virulent over the course of the patient’s infection. A summary of the LD_50_ values and the other characteristics of the five representative hypermutator and non-hypermutator isolates are listed in Table S4.

### Mutations that emerged during infection

We next examined the most common non-synonymous mutations that emerged during the course of the patient’s infection. We reasoned that commonly observed mutations may reflect adaptations that provide a fitness advantage to *P. aeruginosa* in the pulmonary environment or in the presence of antibiotics. Compared to L00-a, all subsequent non-hypermutator and hypermutator isolates contained a non-synonymous mutation in the *lasR* gene, which encodes a regulator of the *las* quorum-sensing system (Table 1). Other genes commonly mutated in both non-hypermutator and hypermutator isolates included *mexR*, *mexB*, *oprD*, and *pvdS* (an alternative sigma factor that regulates the siderophore pyoverdine and other factors). A number of mutations occurred in all hypermutator isolates but not in non-hypermutator isolates, including mutations in *pilB* (biogenesis of type IV pili), *hitA* (ferric iron-binding periplasmic protein), and several uncharacterized genes (Table 1). A complete list of mutations can be found in Table S2. These findings suggest that selective pressures within the airways of infected patients drive adaptations in *P. aeruginosa* and that the hypermutator phenotype may accelerate this process.

**TABLE 1:** Most common non-synonymous mutations observed in the patient’s *P. aeruginosa* isolates^*a*^

		Number of isolates with non-synonymous mutations relative to L00-a	
Gene	Amino acid change	Hypermutators (*n* = 14)	Non-hypermutators (*n* = 7)
Transcriptional regulator LasR (PA1430)	M25K	14 (100%)	7 (100%)
Multidrug resistance operon repressor MexR (PA0424)	G101W	14 (100%)	6 (86%)
Resistance-nodulation-cell division (RND) multidrug efflux transporter MexB (PA0426)	R620C	14 (100%)	6 (86%)
sigma factor PvdS (PA2426)	V71A	14 (100%)	6 (86%)
Basic amino acid, basic peptide, and imipenem outer membrane porin OprD precursor (PA0958)	W339*	14 (100%)	6 (86%)
hypothetical protein (PA4048)	A133V	14 (100%)	0
hypothetical protein (PA3722)	A69G	14 (100%)	0
D-Serine dehydratase (PA3357)	F29L	14 (100%)	0
Putative two-component sensor (PA3044)	R466W	14 (100%)	0
Soluble pyridine nucleotide transhydrogenase (PA2991)	I143V	14 (100%)	0
Nitrate transporter (PA1783)	G381D	14 (100%)	0
Hypothetical protein (PA0830)	Y244H	14 (100%)	0
Hypothetical protein	H114R	14 (100%)	0
Putative transcriptional regulator (PA0791)	R108W	14 (100%)	0
Penicillin-binding protein 3 (PA4418)	R504H	14 (100%)	0
Type IV fimbrial biogenesis protein PilB (PA4526)	V358M	14 (100%)	0
Ferric iron-binding periplasmic protein HitA (PA4687)	G55S	14 (100%)	0
DNA mismatch repair protein MutL (PA4946)	H472L	14 (100%)	0
Putative binding protein component of ABC transporter (PA5076)	T104A	14 (100%)	0
Hypothetical protein	I62V	14 (100%)	0
Porphobilinogen deaminase (PA5260)	D293G	14 (100%)	0

^*a*^ *, premature stop codon.

## DISCUSSION

Hypermutator strains have been associated with increased antibiotic resistance and altered virulence phenotypes in *P. aeruginosa* lineages causing chronic infections, especially those in CF (6, 9, 10, 12). In the case reported here of a patient with repeated bouts of VAP, a *P. aeruginosa* hypermutator lineage emerged over weeks to months rather than years. The non-hypermutator and hypermutator lineages co-existed with each other and developed resistance to several antibiotics. Hypermutator isolates were attenuated in a cell culture and a mouse model of virulence, and one such isolate lost the ability to secrete ExoU, a major *P. aeruginosa* virulence determinant. These results demonstrate that hypermutator strains can emerge over relatively short periods of time in individuals lacking pre-existing structural lung disease. In addition, these lineages may impact virulence, which in turn has the potential to influence patient outcomes.

While hypermutator strains of *P. aeruginosa* have been detected in 10%–54% of individuals with CF (4), they are much less common in acute infections. For example, Oliver et al. (11) did not detect hypermutator strains in *P. aeruginosa* isolates cultured from 50 patients with bacteremia and 25 patients with non-CF respiratory infections or colonization. Similarly, Gutiérrez et al. (14) tested 160 *P. aeruginosa* isolates from 103 non-CF patients and noted only one patient with a hypermutator strain. More recently, Torrens et al. (42) examined 723 *P. aeruginosa* isolates cultured from 402 patients in European intensive care units and found that only two were hypermutators. In a report from the U.S. Centers for Disease Control and Prevention of 1,019 carbapenem-resistant *P. aeruginosa* isolates, 8.1% of isolates from patients without CF were hypermutators (43). Even in CF patients, hypermutators tend to emerge after years of infection rather than subacutely; studies have found only 0%−10% of newly infected CF patients harbor hypermutator strains, whereas 24%−73% of chronically infected CF patients harbor these strains (5, 10, 44). Our patient did not have CF or other known structural lung diseases. The patient did, however, have the same *P. aeruginosa* lineage persist in their lungs for several months, as commonly occurs in individuals with CF. Despite repeated bouts of antibiotics and partial clinical resolution, microbiological eradication was not achieved. This prolonged persistence of *P. aeruginosa* likely provided the opportunity for the bacterium to evolve into the hypermutator phenotype. Among respiratory bacteria that cause acute pneumonia, *P. aeruginosa* is notable for its ability to persist even in the face of active antibiotics (45, 46). Of note, Liu et al. (47) recently reported seven non-CF intensive care unit patients from which *P. aeruginosa* was cultured for 4–19 months. In two of these patients, hypermutators emerged. Our findings demonstrate that hypermutator strains of *P. aeruginosa* may indeed arise in the setting of acute or subacute infections in the absence of pre-existing structural lung disease.

The co-existence of both hypermutator and non-hypermutator isolates throughout most of the patient’s infection was noteworthy and suggested that different selective pressures were favoring each lineage. This in turn suggested that reversions from hypermutator to non-hypermutator genotypes may have occurred. To investigate this, we examined the *mutL* gene in each of our isolates but did not detect evidence of reversions. Likewise, isolates with mutated *mutL* alleles but only a small number of mutations were not observed. Such a finding would have been consistent with the reversion of hypermutator status via a second site suppressor mutation early after the occurrence of the initial *mutL* mutation. The absence of both of these findings suggests that reversions are rare but do not exclude their occurrence.

*P. aeruginosa* is notorious for its ability to develop resistance to antibiotics during the course of therapy, which occurs in approximately 10% of patients (48, 49). The patient reported here illustrates this phenomenon. Resistance to some carbapenems was observed even prior to the apparent emergence of the hypermutator lineage (Fig. 4). Further loss of susceptibility to other antibiotics was observed later in infection and involved high levels of resistance to cefepime and ceftazidime and intermediate susceptibilities to aztreonam, levofloxacin, and piperacillin-tazobactam. However, during this same time, the hypermutator lineage acquired additional high-level resistance to aztreonam, levofloxacin, ciprofloxacin, and gentamicin. These findings illustrate that antibiotic resistance testing of a single isolate may provide misleading information regarding the selection of appropriate antimicrobial therapy for a patient.

Our findings demonstrate ways in which hypermutation may lead to changes in virulence. One of the hypermutator isolates lost the ability to secrete ExoU, a type III secretion effector protein that has been associated with worse outcomes in *P. aeruginosa* infections in both experimental models and human infections (34, 50). Although examination of more patients and isolates is necessary, this observation suggests that the increased difficulty in treating antibiotic-resistant hypermutator infections may be somewhat offset by a decrease in the intrinsic virulence of these strains.

One interesting aspect of our findings is that several mutations that emerged in the *P. aeruginosa* isolates from our patient also commonly occur in CF infections. For example, most of our isolates contained mutations in *lasR*, *mexR*, *mexB*, *oprD*, and *pvdS*, all of which are frequently observed in isolates from individuals with CF (51–53). This suggests that the fitness pressures driving the emergence of these mutations are not specific to CF but rather are the result of antibiotic therapy or the pulmonary environment in general. A remaining question is how long *P. aeruginosa* must be exposed to these fitness pressures before these mutations emerge.

Together, these findings suggest that *P. aeruginosa* hypermutator strains may emerge during acute and subacute infections in the absence of pre-existing structural lung disease and have the potential to affect virulence. Additional studies are necessary to determine how commonly hypermutators arise following VAP and other acute or subacute infections.

## Data Availability

Sequences were deposited in the National Center for Biotechnology Information (NCBI) database (see Table S1 for accession numbers). Custom software scripts are available at the links listed in the text.

## SUPPLEMENTAL MATERIAL

Table S1 msystems.00484-23-s0001.xlsx WGS information.

Table S2 msystems.00484-23-s0002.xlsx SNVs.

Table S3 msystems.00484-23-s0003.xlsx OSH MICs.

Table S4 msystems.00484-23-s0004.xlsx Summary of isolates.

ASM does not own the copyrights to Supplemental Material that may be linked to, or accessed through, an article. The authors have granted ASM a non-exclusive, world-wide license to publish the Supplemental Material files. Please contact the corresponding author directly for reuse.
